# The rare disease burden: a multidimensional challenge

**DOI:** 10.3389/abp.2025.14777

**Published:** 2025-07-14

**Authors:** Zuzanna Cyske, Edyta Radzanowska-Alenowicz, Estera Rintz, Lidia Gaffke, Karolina Pierzynowska

**Affiliations:** ^1^ Department of Molecular Biology, Faculty of Biology, University of Gdansk, Gdansk, Poland; ^2^ Faculty of Social Sciences, University of Gdansk, Gdansk, Poland

**Keywords:** rare diseases, molecular causes of diseases, diagnostics, treatment, multidisciplinary research, multiple challenges

## Abstract

Although there no single, widely accepted definition of a “rare disease,” this group of disorders includes conditions that affect only a small fraction of the population. A large number of rare diseases is caused by defined molecular defects, predominantly the occurrence of pathogenic variant(s) of genes. Thus, they are classified as “genetic diseases,” among which there are many neurodegenerative disorders. Despite a low incidence of each such disease, majority of them are severe and no effective treatment is available. This creates a battery of problems for millions of people suffering from these disease as well as to their relatives and caregivers. However, the set of problems is larger; therefore, in this narrative review we summarize and discuss various kinds of problems caused by rare disease, including severe symptoms of patients, everyday problems of patients and caregivers, loneliness and social exclusion, diagnostic difficulties, unavailability of effective therapies and economic difficulties in introducing orphan drugs, and a complexity of studies on rare diseases due to low availability of biological material and complicated pathomechanisms. The global picture of the complex problems caused by rare diseases is presented.

## Introduction

There is no single, specific, and commonly accepted definition of a rare disease, as each country/region of the world has established its own criteria for classifying a disease as rare or ultra-rare (Hughes et al., 2005; Richter et al., 2015; Kölker et al., 2022; Song et al., 2013). For example, according to the World Health Organization (WHO), a rare disease is one that affects no more than 1 in 2,000 people (The Lancet Global Health, 2024). In European Union, the definition is similar, but expressed as less than 5 in 10,000; however, in United States the definition describes the total number of patients, rather than a frequency, and it is expressed as less than 200,000 in the population of the country (which corresponds to about 1 in 1,500 people). Regardless of the specific definition, it is estimated that nearly 10,000 rare diseases have been identified to date, affecting approximately 6% of the global population - around 400 million people. (last accessed on 28 February 2025^1^). Taken globally, these numbers indicate that the term “rare” is no longer correct. Hence, it is very important to constantly seek and discover innovative therapeutic methods, effective in eliminating or at least reducing the number of “rare uncurable diseases,” even despite the high costs of introducing the drug to the market.

When considering etiology, it is estimated that about 80% of rare diseases are of genetic origin, although for some of them the specific genetic cause has not yet been identified (last accessed on 28 February 2025^1^). This means that for most of them, specific pathogenic variant(s) of a specific gene or a broader genetic defect (like aneuploidy, large deletions of chromosomes, chromosomal translocations, and others) are known. Nevertheless, the molecular mechanisms underlying monogenic diseases are often complex, as the dysfunction of a single gene, and resulting defect in one protein, affects a complex network of biochemical reactions and their cellular consequences. In fact, there is no specific treatment available for vast majority (over 95%) of rare diseases.

Because of the genetic character, symptoms of most of rare diseases are expressed in a childhood. Another problematic characteristics is that neurodegenerative disorders occur in some 70% of rare diseases. Neurodegenerative diseases are currently the seventh leading cause of death worldwide (Hughes et al., 2005).

An mentioned above, the challenges associated with ‘rare diseases’ are complex and require systematic actions improve the quality of life for both patients and their caregivers. To overview this broad topic, and to indicate common issues specific to most “rare diseases,” this article will overview and discuss the problems affecting people with these diseases from several perspectives, including (i) problems faced by patients and their parents/caregivers (as mentioned earlier, most patients are children); (ii) problems with making a faster and accurate diagnosis, i.e., problems faced by physicians diagnosing patients, which is often crucial to ensure maximal possible quality of life; (iii) problems with developing and introducing to the market a therapy available to every patient, which consist of problems faced by researchers working on understanding the disease pathomechanism, finding molecular targets of potential drugs, and developing potential therapies, as well as of introducing specific drugs to the market due to economic constrains and barriers.

## Methods

This is a narrative review, presenting opinions and experiences of the authors. Literature search included analysis of the PubMed database (last accessed on 28 February 2025^2^), using the term “rare disease.” However, since this search gave a huge number of 392,129 records, a strong selection of papers, strictly related to the subject of this review and cited here, was performed. Moreover, some other Internet sources, related to “rare disease” were considered, including Global Genes – Allies in Rare Disease (last accessed on 28 February 2025^1^), American Academy of Pediatrics (last accessed on 28 February 2025^3^), Rapsody Online (last accessed on 28 February 2025^4^), and the European Union Rare Disease Patients Solidarity Project (last accessed on 28 February 2025^5^).

## Challenges of patients

Patients suffering from rare diseases face numerous challenges, significantly more than those diagnosed for more common disorders. The primary issue is that due to usually complicated molecular mechanisms and complex changes at cellular and organismal levels, the symptoms are severe and often worsen over time. Therefore, increasing suffering each year occurs in many diseases from this group and, in some cases, there is a need for round-the-clock care.

A classic example is children with mucopolysaccharidosis (MPS),a group of inherited metabolic diseases caused by defects in genes coding for enzymes responsible for degradation of glycosaminoglycans which, in turn, accumulate in the cells. MPS patients experience a wide variety of symptoms due to the vast heterogeneity of the disease, including skeletal deformities, intellectual disabilities, respiratory issues, and sleep disturbances. Over time, these symptoms intensify, and additional complications emerge, further hindering daily functioning (Rintz et al., 2024). For many years it was believed that the only cause of MPS is the accumulation of GAGs in lysosomes, but over the years it has been shown that this is not the only problem in the pathogenesis of the disease. It has been proven that in addition to the accumulation of GAGs, the problem is also the reduced efficiency of cellular processes. It is therefore possible that it is not the appearance of GAGs alone, but the reduced efficiency of key molecular processes taking place in cells that is responsible for the pathogenesis of the disease (Pshezhetsky, 2015; Tessitore et al., 2009). In contrast, in patients with Huntington disease, symptoms appear much later, but just as with other rare diseases, they eventually prevent independent functioning, and over time, lead to exclusion from professional and social life (Doss et al., 2025). An example might be spinocerebellar ataxia (SCA), a disease that is extremely diverse, and its onset can manifest in both small children and adults. Recent studies have shown that one of the causes of this disease may be a mutation in the *FGF14* gene (Hirschfeld AS et al., 2025).

Research aimed at developing a cure for any disease is possible due to the constantly developing diagnostic/molecular methods; without this progress it would not be possible. For example, sequencing methods allow finding different variants of diseases or allow identifying a specific disease in patients who often remain undiagnosed for many years.

Children and adolescents with rare diseases encounter difficulties in adapting to the traditional education system. Doctor visits, hospitalizations, and health restrictions affect or prevent school attendance. This often leads to exclusion from peer groups. As a result, there are difficulties in building social relationships and maintaining motivation to learn (Song et al., 2013; Rintz et al., 2024).

People with rare diseases can feel isolated, both socially and emotionally. Because these diseases are little known, society can lack understanding and empathy for the patient’s suffering. Patients with rare disease often struggle to maintain social connectionswith healthy individuals, as their daily lives differ significantly. As a result, they can feel isolated, and their health challenges can be overlooked by those around them (Lopes et al., 2018; Ramalle-Gómara et al., 2015).

Children growing up in families where one member has a rare disease may also experience difficulties. On the one hand, siblings may feel neglected because parents focus most of their attention on the child affected by illness. This can lead to feelings of rejection and lack of emotional support. On the other hand, siblings may feel pressured to be “good,” “understanding” or “helpful,” often at the expense of expressing their own emotions or needs. They may become jealous of their parents’ attention or feel less important (Flores et al., 2022; Desser, 2013; Spencer-Tansley et al., 2022). Rare diseases, especially those with visible symptoms, can lead to stigmatization. In the case of diseases that cause changes in appearance (e.g., body deformities), patients may experience negative social reactions, such as avoidance, ridicule, or worse treatment. Stigmatization can also affect people with diseases that affect the patient’s behavior (e.g., neurological disorders), which can lead to rejection by peers, colleagues, or even family members. Adults with rare diseases can face many challenges in securing and maintaining employment. Their health conditions can limit their career options, and in some cases, their illness can require frequent breaks from work, making employers reluctant to hire them. Additionally, a lack of understanding from coworkers can lead to isolation in the workplace, which can lead to low self-esteem and fear of rejection (Spencer-Tansley et al., 2022).

Access to specialist doctors, who can provide an accurate diagnosis, is another significant issue for patients. As a result, many remain undiagnosed for an extended period. This problem may also be linked to the insufficient number of physicians specializing in rare diseases. The issue is highly complex, as the vast number of rare diseases necessitates extensive research, which can be both time-consuming and costly. Accurate and timely diagnosis is essential for initiating treatment and mitigating the impact of the disease, even if no specific cure is currently available. Unfortunately, even after a proper diagnosis is made, the problem does not disappear. Patients often begin searching for the best possible treatment, which is not always available to them for various reasons, including a lack of specific treatment or high costs or therapies offered (Lopes et al., 2018). Prenatal testing of genetic defects is also an option, but most standard tests do not detect all mutations; they tend to focus on the most common ones. More specific tests are required for an accurate prenatal diagnosis of rare diseases, and prospective parents usually do not undergo such tests without a clear reason, such as a previous child with the disease or information of being a carrier (Ramalle-Gómara et al., 2015; Flores et al., 2022; Desser, 2013).

The most obvious and most important effect of the disease is the enormous suffering of the patient, not only because of the pain, but also for psychological reasons. The patients, if not developing severe cognitive deficits, are aware that they may not receive effective treatment and, consequently, that they will not recover. This can lead to a serious deterioration of their mental health and, over time, to the development of depression (Spencer-Tansley et al., 2022; Uhlenbusch et al., 2019). It have been shown that patients with rare diseases and their caregivers experience enormous, everyday anxiety, stress, anger or frustration, not only due to the symptoms of the disease, but also due to other aspects of life. The deteriorating mental health was associated with the fear of how the disease may affect their work and studies, relationships with partners, peers, friends or relatives (Spencer-Tansley et al., 2022). These studies have shown that 36% of patients and 19% of caregivers had suicidal thoughts.

Because of the rarity of these diseases, individuals affected by them often struggle to find others facing similar challenges. The lack of support groups that offer emotional and practical support makes it difficult to share experiences and understand the problems associated with the disease. A community of patients can be crucial in getting through difficult times, and its absence can lead to feelings of loneliness. Obviously, there are many global and local organizations that specialize in helping patients in various areas of their lives, financial, psychological, medical help, but also support in everyday functioning (shopping, cleaning, medications). Moreover, foundations can be helpful, as in addition to providing daily help to patients, they organize many conferences where patients and their parents/guardians can learn about the disease that has affected the family. This is often the only source of information, due to the limited number of scientific publications and especially popular science articles. Another help for patients is the possibility of contact with other patients, with foundations and associations via the Internet, and also obtaining information on reliable and verified websites (like^6,^ ^7^) where one can find useful and interesting information. Patients and/or their families can also learn about the symptoms, course of the disease of interest or causes of the disease. A very interesting initiative was launched in 2008. Every year in February, a rare disease day is organized. Gradually, the event gained a global rank and is now organized in over 100 countries. The formal date of the Rare Disease Day is 29th February (as this is the rarest day in a calendar), but in years when 29th February does not occur, the Day is celebrated on 28th February.

Another important aspect involves the challenges faced by doctors caring for patients with rare diseases. Patients seeking help reported problems with difficulty in making a diagnosis, which resulted from insufficient knowledge of physicians about rare diseases. Most patients, even after making an accurate diagnosis and starting treatment, continue to live with great anxiety related to difficulties in finding prescribed medications (Richardson et al., 2024). In some instances, healthcare providers failed to take the patient’s symptoms and concerns seriously, occasionally viewing them as a mere “curiosity” (Spencer-Tansley et al., 2022; Richardson et al., 2024). A significant stress factor turned out to be the lack of understanding of the disease on the part of society and the need to constantly explain and explain the disease to others. A huge problem may be the exclusion of a sick child from social life. Due to insufficient knowledge or misunderstanding of the problem, the child may be rejected by peers (Spencer-Tansley et al., 2022), and may even be accused of excessive laziness (Lopes et al., 2018). In such a case, a quick reaction on the part of caregivers/teachers may be of great importance. However, for this to be possible, the caregivers/teachers themselves should be properly prepared and trained for this.

A major challenge, sometimes even preventing treatment, may be the physical or geographical distance. Patients are often scattered all over the world, while clinical trials and specialists with the best possible knowledge of a given disease entity can be located in only one, often very distant research centre. Distance, costs related to travel and accommodation at the place of treatment, the patient’s health, work and often many other factors can prevent treatment. Furthermore, even if treatment exists for a given disease, it is often associated with frequent visits to a specific hospital that has the medicine, necessitating travel not only for the patient but also for their caregiver (Cacoub et al., 2025).

## Challenges of parents/families/caregivers

The illness of a child is always a tremendous tragedy for the parent, and additional challenges arise when a rare disease is diagnosed, for which there is often no effective treatment (Spencer-Tansley et al., 2022). In such cases, most parents attempt to find therapies on their own that might save their child’s life. Even when a treatment for a specific disease is developed, it is often prohibitively expensive, and parents do not always have sufficient financial resources (Song et al., 2013). An additional complication is the fact that, in most cases, the child will need to take medication for many years. This brings about another serious issue: due to frequent doctor visits or rehabilitation sessions, parents are often forced to reduce or even eventually give up their paid employment. As a result, their income decreases, making it increasingly difficult to finance their child’s long-term treatment. In fact, a need for complex care on both patients and parents have been recognized, and presented employing a comprehensive analysis of the case of one, selected rare disease (though the problems are definitely common for most rare diseases) (Cyske et al., 2022).

An additional and serious problem may also be the possibility of the same rare disease occurring in the younger siblings of the patient. In such a case, parents should start diagnostics as soon as possible, because introducing therapy before the first symptoms appears to give agood chance that they will not develop at all, if a specific therapy is available. Nevertheless, even if no cure can be proposed now, a complex care may result in a significantly better quality of life of both patients and their parents. According to studies conducted in Great Britain, the mental health of parents of sick children deteriorates due to the stress associated with constant worry about the health and wellbeing of their children (Spencer-Tansley et al., 2022). Additionally, parents may also have problems with obtaining sufficient information about their child’s disease and possible methods of treatment (Desser, 2013). Again, this highlights the need for early diagnosis and further medical treatment (even if symptomatic or palliative) which is, however, often difficult (Wiśniewska et al., 2022).

Many people are unaware of the existence of rare diseases, which leads to misunderstandings and stereotypes. The lack of public knowledge about the specifics of these diseases can cause people affected by them to be treated differently, which increases their sense of exclusion, and results in the pressure to parents. Educating the public about these diseases is key to reducing stigma and supporting patients and their families in integrating into society. The role of patient organizations, as well as support and advocacy groups is not to be underestimated, as their activities appear to be crucial to ensue quite normal existence of patients and their families (Taruscio and Gahl, 2024).

Families, especially parents, may have to take on the role of full-time caregivers, which is associated with many difficulties. Caring for someone with a rare disease requires a huge investment of time and energy, especially since patients need regular doctor visits, therapy and specialist treatment. This affects the daily life of the family and especially the professional life of the parents. As a result, one of the parents, most often the mother, has to limit his or her professional life, leading to loss of income and additional financial burden. As a result of the constant responsibilities associated with caring for a sick family member, parents may have difficulty balancing their personal lives with their caring responsibilities. This can lead to neglect of the relationship between partners, which increases the risk of conflict and even the breakdown of marriages. Financial and emotional problems, as well as a chronic lack of time, which means that parents lack the strength to nurture their relationship, which leads to a sense of loneliness, the loss of relationships and often the breakdown of the relationship. In many countries, social care systems may not be adequately prepared to support people with rare diseases. The lack of adequate health, psychological and social services can make patients feel neglected and unable to access appropriate care. Furthermore, access to specialist care is often limited, especially in less developed regions, which increases inequalities in access to treatment.

## Challenges of physicians

A major challenge in diagnosing rare diseases is forming the correct hypothesis about the type of disease, identifying the right research centre, and, most importantly, finding a doctor capable of making an accurate diagnosis and providing effective treatment. Unfortunately, when a medical facility or research center lacks specialization in a specific disease, the chances of achieving an accurate diagnosis are significantly reduced. This is mainly due to the fact that there are a huge number of rare diseases - at least several thousand - making it difficult for any single physician to be familiar with all of them. Studies conducted in Spain and Peru showed that doctors have limited knowledge of rare diseases (Lopes et al., 2018; Ramalle-Gómara et al., 2015; Flores et al., 2022). The same studies have shown that physicians are concerned about the insufficient education of future doctors on the subject of rare diseases (Lopes et al., 2018; Flores et al., 2022). On the other hand, surveys conducted among Norwegian and Spanish doctors have shown that they give much higher importance in financing diseases that are more common in society than diseases from the rare group (Desser, 2013). This is understandable to some extent, because it is obvious that a much larger part of the population suffers from such diseases, but does it mean that one of these conditions is more important, more significant, more painful for the patient?

The issue of dividing funds between rare and common diseases is certainly complicated by the fact that the treatment of people with rare diseases is less common, but the treatment is much more expensive, while the opposite may be true for more common diseases (Flores et al., 2022; Cacoub et al., 2025). The lack of interest in treating rare diseases may be the result of insufficient knowledge about the effects of this group of diseases. Another huge problem may be the failure to adapt diagnostic tests to the needs or age of the patient. Such difficulties have been reported by doctors dealing with Krabbe’s disease, which manifests itself in problems with the central nervous system. The first symptoms usually appear in the first year of life, so a doctor using most standard diagnostic tests will not be able to diagnose the disease (Richardson et al., 2024). This will consequently lead to a delay in diagnosis and loss of valuable time. Advanced screening methods, like multi-omics and AI-driven analyses, can expand genetic disease detection, especially in underserved areas. Early diagnosis enables timely intervention and identifies cases with mild or atypical symptoms. Combining broader screening with genotype-phenotype insights helps clinicians diagnose, assess severity, and personalize treatment more effectively (Gallagher, 2024).

## Challenges of researchers

Researchers studying rare diseases face many challenges. Unfortunately, the world of science is very closely linked to money, and obtaining funding for such research is very difficult and often involves rejection. Additionally, due to the difficult economic situation, institutions financing research often limit the overall budget for grants which makes the possibility of receiving it incredibly small. Even if a project falls within the scopeof possible funding, most reviewers often prioritize proposals perceived to have “greater impact” - typically studies involving larger numbers of cell lines, animals, or patients, and focusing on more prevalent conditions, such as common diseases. Research aimed at developing a drug is extremely time-consuming, because it begins with research on cells (*in vitro*), and only after a positive conclusion of this phase of experiments can one move on to research to determine the effect of a potential drug on the entire organism, i.e., animal research. Only such long-term, time-consuming and expensive research allows for further research, including clinical trials. However, each of these stages has its limitations. At the very beginning, scientists face the availability of cell lines for *in vitro* research. As mentioned, most rare diseases affect children, thus parents first face the difficulty of finding a specialist who will make an accurate diagnosis. Then they fight for the best possible therapy, in the meantime struggling with many other problems, including financial, social, and psychological ones. The last thing they will think about, if at all, will be depositing cells in cell banks, from which scientists can buy them in order to conduct research and potentially develop a therapy. The next stage of research, animal experiments, also require a lot of time, knowledge, and money. This involves the need to obtain many consents to carry out procedures on animals, including the consent of the Local Bioethics Committee. The person submitting such consent must have knowledge and experience in working on animals. Finding an institution that will decide to finance research on rare diseases and will have sufficient funds can also be a huge difficulty.

Finding an institution that will decide to fund research on rare diseases and will have sufficient financial resources can also be a huge obstacle. Scientists often work with many foundations and associations that organize all kinds of help for patients, including trying to fund research that may lead to the creation of drugs. Most countries have research funding centers in various fields, but they prefer research that has a larger audience, which means that getting funding for a rare disease is extremely difficult. Fortunately, in recent years, more and more institutions/projects have been established that fund research on rare diseases (e.g., ERDERA^8^; The NORD^9^). Because they focus mainly on rare diseases, there is a greater chance of receiving a grant. Moreover, “N = 1 Collaborative”^10^ is an organization that brings together patients, their families, scientists, doctors, physiotherapists, founders and many others who work together for a better future for patients. The organization focuses on providing patients with rare diseases with access to individualized treatment, tailored to the needs of a specific patient at a specific moment in the development of the disease^10^.

Innovative gene editing methods (like CRISPR/Cas9) may offer a great hope for patients, as they enable the manipulation of a given organism’s gene. The use of this method in the treatment of patients was first approved in 2023 in the UK for the therapy of sickle cell anemia and thalassaemia (Cetin B et al., 2025). Another similar and equally promising discovery of recent years concerns antisense oligonucleotide therapy. Antisense oligonucleotides (ASOs) are short oligonucleotides that can affect the modification of gene expression. These molecules show potential in the therapy of genetic diseases (Lauffer MC et al., 2024).

## Clinical trials

The process of introducing a new drug to patients begins long before clinical trials begin. The necessary first stage is research on cell models to determine the effect of the potential drug on a cell function, cellular viability and safety. Then the research moves on to the next phase, i.e., studies aimed at determining the effect of the drug on the entire organism, i.e., animal research. Only after obtaining positive results from these experiments can clinical trials involving patients begin. Unfortunately, pharmaceutical companies tend to prioritize funding forresearch with a broader scope, i.e., research on drugs that affect a larger number of people in the world, rather than on rare diseases. This approach allows them to maximize profits. However, as a result of such company policies, patients suffering from rare diseases have very limited, and sometimes even impossible, access to treatment. Nevertheless, this is not a malice or reluctance of pharmaceutical companies, but rather a sole economic constraint. One should take into consideration that developing a drug for a rare disease, so called “orphan drug,” is as costly as in the case of any other disease, however, number of patients which can potentially buy it is very low. Therefore, an orphan drug must be very expensive, not necessarily because of a high costs of its production, but rather because low number of potential buyers who might purchase such a product. This makes a business related to developing orphan drug very risky economically for pharmaceutical companies. One potential solution would be to develop one drug for many diseases (Pierzynowska et al., 2020). However, it is clear that it would not always be possible, thus, the problem awaits also other solutions.

Clinical trials offer hope for patients, but strict eligibility criteria often prevent many from participating, leaving them without treatment options. The situation has also been made more difficult by the introduction of many restrictions aimed at ensuring patient safety and demonstrating the highest possible effectiveness of the drug being introduced (Haffner et al., 2002; Haffner, 2006). It is obvious that such studies are absolutely necessary to ensure patient safety, but unfortunately the introduction of such restrictions caused pharmaceutical companies to decide to discontinue research on orphan drugs, because it was not profitable for them due to the small patient population (Wästfelt et al., 2006). However, in order to encourage pharmaceutical companies to develop drugs for rare diseases, the Orphan Drug Act was introduced, which gives a company the right to market exclusivity for 7 years from the introduction of the drug, tax relief and exemption from fees (Melnikova, 2012; Meekings et al., 2012). It can therefore be observed that the introduction of such facilities encouraged companies to conduct clinical trials on drugs for rare diseases, because since the entry into force of the Act, over 400 drugs for rare diseases have been approved, while only 10 before the introduction of the Act (Melnikova, 2012; Kumar Kakkar and Dahiya, 2014).

## Costs of treatments

Pharmaceutical companies that develop treatments for a rare disease to the market usually has exclusivity for it, enabling them to set prices with minimal competition. In addition, the patient has no other alternative if they want to start treatment, and they also have no opportunity to negotiate. In fact, the price they have to pay must cover the cost of introducing the drug to the market, i.e., the cost of all tests, starting with *in vitro* tests (Kumar Kakkar and Dahiya, 2014). That is why the costs of treating rare diseases are so huge, as explained in the preceding section (Simoens, 2011).

Looking at examples of several diseases separately, the cost of annual treatment of cystic fibrosis is 300,000 dollars (EvaluatePharma, 2017), Gaucher disease - 400,000 dollars per year (O'Sullivan et al., 2013), paroxysmal nocturnal hemoglobinuria - 500,000 dollars per year (Meekings et al., 2012). It have been estimated that the average direct cost of introducing a new drug may amount to about 1.4 billion dollars, while taking into account the capitalization of costs, the total amount may be about 2.6 billion dollars (Haffner et al., 2008). However, other estimates indicated considerably lower amounts, i.e. 757 million dollars (capitalized costs) for introducing a cancer drug to the market (DiMasi et al., 2016). In this light, it is worth mentioning the estimation showing that a drug registered for a rare disease is about 8 times more expensive than a drug for a more common disease (Simoens, 2011). Obviously, it also depends on whether more drugs or only one are registered for a given disease. Several drugs are currently registered for renal cell carcinoma and cystic fibrosis, thus the price may also be correspondingly lower (Prasad and Mailankody, 2017).

Differences in prices may also result from different policies of countries approving the treatment (Simoens, 2011). Nevertheless, generally prices of orphan drugs are extremely high which in most cases precludes their purchasing by individual patients. The only possibility is to count on reimbursement from either health insurance companies (if appropriate policy is active, which is usually problematic due to relatively high costs of policies covering costs of treatment of rare diseases) or governmental agencies; the latter possibility differs significantly from country to country and even in the same country the rules are changes frequently (Pierzynowska et al., 2020; Simoens, 2011; O'Sullivan et al., 2013; Dear et al., 2006).

## Social assistance

As mentioned earlier in this article, many rare diseases are progressive, gradually limiting or eventually eliminating the patient’s ability to live independently. Patients’ families often do not have sufficient knowledge and/or skills to fully help the patient, which can lead to even greater harm. In such cases, specialist help is necessary in many ways. It is necessary for the doctor to select the appropriate, personalized therapy for the needs of the patient, for the physiotherapist to be trained to work with patients suffering from various disorders, and for the psychologist to know whether and when to introduce appropriate therapy. As mentioned earlier, very often, in addition to physical problems, patients also have mental problems resulting from misdiagnosis or lack thereof, isolation, pain or misunderstanding by others (Spencer-Tansley et al., 2022; Uhlenbusch et al., 2019). In the United States, nursing homes for people called medical homes have been established, which are to provide basic healthcare, education and support for the patient and their entire family (last accessed on 26 February 2025^3^). Similarly, the European Union has developed the Rare Disease Patient Solidarity Project (RAPSODY), a program that enables better access to care, information and social support for people with diabetes (Bavisetty et al., 2013;^4^ last accessed on 28 February 2025).

Despite the actions described above, social assistance for patients and parents/caregivers suffering from rare diseases while very necessary is still severely underdeveloped, especially in some countries. On the other hand, such assistance is usually crucial to keeping the quality of life of patients at the acceptable level, and to help parents/caregivers to avoid social distancing or exclusion.

## Discussion

**FIGURE 1:**
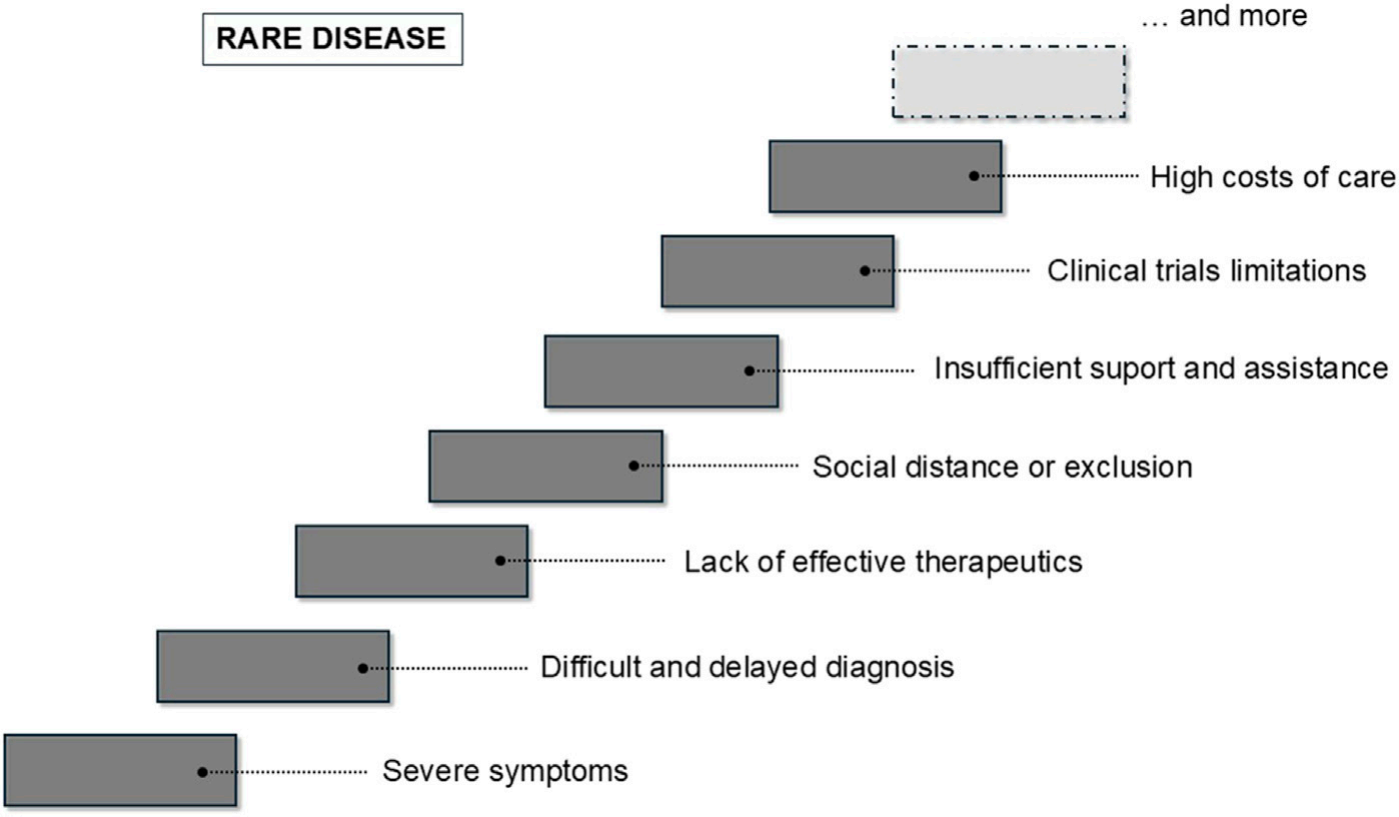
Problems related to rare disease, experienced by patients, parents/families/caregivers, physicians, and researchers.

This article overviews major problems caused by rare diseases. These include not only dramatic problems of patients themselves related to severe symptoms of these diseases, but also problems of parents/caregivers, physicians, and researchers working in this field. Specific problems related to unusual conditions of clinical trials with rare diseases, extremely high costs of treatment, and necessary social assistance are also crucial. The summary of these problems is presented in Figure 1. This provides a picture of how complicated the problem of rare disease is and how complex care is required to provide patients and their families the minimal acceptable level of the quality of life.

On the other hand, the problem is global rather than minor, as there are some 400,000 million people suffering from rare disease, making the problems important not only medically and socially, but also economically (both patients and caregivers are often excluded from professional activities, causing serious economic losses). Ninety-five percent of patients with rare diseases lack available treatments, and without a fresh approach to therapy development, this is unlikely to change. Addressing this issue requires more than just financial support - it demands a shift in strategy and mindset to bridge the gap between clinical care, research, and innovation. Rare diseases pose a significant challenge to both healthcare systems and social policies. In response to these challenges, the European Un-ion has developed the Rare Disease Patients Solidarity Project (last accessed on 28 February 2025^5^). The project focuses on improving access to diagnostics and treatment, developing and spreading knowledge about rare diseases, supporting scientific research. Facilitating access to modern diagnostic methods and therapies, including medicines and special nutritional products, educating society and medical professionals about rare diseases, and financing and coordinating research on new methods of treatment and diagnostics are the main goals of this project.

## Footnotes

1
https://globalgenes.org/rare-disease-facts/

2
https://pubmed.ncbi.nlm.nih.gov/

3
https://www.aap.org/en/practice-management/medical-home

4
http://rapsodyonline.eurordis.org/

5
https://health.ec.europa.eu/social-determinants/overview/commission-communication-solidarity-health-reducing-health-inequalities-eu_en

6
www.orpha.net

7
www.omim.org

8
http://erdera.org

9
http://rarediseases.org

10
https://www.n1collaborative.org/
